# Synergistic Re-Activation of Epigenetically Silenced Genes by Combinatorial Inhibition of DNMTs and LSD1 in Cancer Cells

**DOI:** 10.1371/journal.pone.0075136

**Published:** 2013-09-06

**Authors:** Han Han, Xiaojing Yang, Kurinji Pandiyan, Gangning Liang

**Affiliations:** 1 Department of Pharmacology and Pharmaceutical Sciences, School of Pharmacy,University of Southern California, Los Angeles, California, United States of America; 2 Departments of Urology and Biochemistry and Molecular Biology, Norris Comprehensive Cancer Center, Keck School of Medicine, University of Southern, California, Los Angeles, California, United States of America; 3 Program in Human Genetics, Johns Hopkins School of Medicine, Baltimore, Maryland, United States of America; Peking University Health Science Center, China

## Abstract

Epigenetic gene silencing, mediated by aberrant promoter DNA hypermethylation and repressive histone modifications, is a hallmark of cancer. Although heritable, the dynamic nature and potential reversibility through pharmacological interventions make such aberrations attractive targets. Since cancers contain multiple epigenetic abnormalities, combining therapies that target different defects could potentially enhance their individual efficacies. 5-Aza-2'-deoxycytidine (5-Aza-CdR), FDA-approved drug for the treatment of myelodysplastic syndrome, can inhibit DNA methyltransferases (DNMTs) upon incorporation into the DNA of dividing cells, resulting in global demethylation. More recently, the first histone demethylase, lysine specific demethylase 1 (LSD1), which demethylates both histone and non-histone substrates, has become a new target for epigenetic therapy. Using, clorgyline, an LSD1 inhibitor (LSD1i) to treat cancer cell lines, we show that clorgyline employs two mechanisms of action depending on the cell type: it can either induce global DNA demethylation or inhibit LSD1-driven H3K4me2 and H3K4me1 demethylation to establish an active chromatin configuration. We also investigate the therapeutic efficacy of combining 5-Aza-CdR with clorgyline and determine that this combinatorial treatment has synergistic effects on reactivating aberrantly silenced genes by enriching H3K4me2 and H3K4me1. Many of the reactivated genes are categorized as cancer testis antigens or belong to the interferon-signaling pathway, suggesting potential implications for immunotherapy. Together, our results demonstrate that combinatorial treatment consisting of a DNMT inhibitor (DNMTi) and an LSD1i have enhanced therapeutic values and could improve the efficacy of epigenetic therapy.

## Introduction

Gene silencing mediated by aberrant promoter DNA hypermethylation and histone modifications is one of the hallmarks of cancer. Although such modifications are heritable, their dynamic nature and reversibility through pharmacological interventions make them attractive therapeutic targets [1]. Over the past few decades, various drugs that target different types of epigenetic alterations have been developed with the goal of reactivating aberrantly silenced genes, including DNMTi and histone deacetylases inhibitors (HDACi) [2], [3]. Many of them have shown promising therapeutic value in treating various malignancies, both as single agents and in combination with other therapies and several have been approved by the FDA.

The N-termini of histones undergo a variety of post-translational modifications to generate transcriptionally permissive or refractory chromatin conformations depending on the type and location of the modification [4], [5]. For instance, transcriptionally active promoters are marked by the enrichments of dimethylation and trimethylation of H3K4 and acetylation of H3 [6]. Transcriptionally inactive promoters are marked by the enrichments of either trimethylation of H3K9 or trimethylation of H3K27 [5]. The balanced activity of histone modifying enzymes that add or remove specific modifications is critical for normal cell physiology [2]. Cancer cells often lack this balance and exhibit a global reduction in acetylation and promoter-specific reduction in di- and trimethylation of H3K4, resulting in aberrant gene silencing [1], [7]. Histone lysine methylation was regarded as a relatively permanent modification until the discovery of the first histone demethylase -lysine specific demethylase 1 (LSD1/KDM1/BHC10/AOF2) [8], [9]. After that, many efforts have been invested in developing inhibitors against histone demethylases.

LSD1 demethylates mono- and dimethylation of H3K4 through a flavin adenine dinucleotide (FAD) dependent mechanism [9], and thus, has the potential to repress gene expression [10]. Prior to the discovery of its demethylating ability, LSD1 was known to associate with a number of co-repressor complexes, including CoREST [11], CtBP [12] and a subset of HDAC complexes [13]. During the demethylation process, an imine intermediate is formed which is further hydrolyzed to generate an unmethylated lysine and formaldehyde as a byproduct [9], [14], [15]. LSD1 can also demethylate a number of non-histone substrates, such as DNMT1, which reportedly makes it more stable [16], [17], thus potentially contributing to increased global DNA methylation. Taken together, LSD1 has two potential mechanisms of action to suppress gene expression: it can demethylate mono- and dimethylated H3K4 as well as stabilize DNMT1.

Overexpression of LSD1 has been reported in a number of malignancies, including acute myeloid leukemia (AML) [18], neuroblastoma [19], breast cancer [20], bladder carcinoma, small cell lung cancer and colorectal carcinomas [21], suggesting that LSD1 inhibitors may have important therapeutic benefit in numerous tumors. LSD1 has been identified to block differentiation in MLL [22] and regulate epithelial-mesenchymal transitions (EMT) to activate motility genes [23]. LSD1 inhibitors can promote differentiation of high grade prostate cancer cells [24], suppress bladder cancer cell proliferation [25], and reactivate aberrantly silenced genes [26]. The catalytic domains of LSD1 and monoamine oxidases share structural homology and make use of the same catalytic mechanism [9]. Therefore, many monoamine oxidase inhibitors are also LSDi. One such monoamine oxidase inhibitor, clorgyline, can also inhibit lysine specific demethylases [19], [20], [27], [28].

Due to the presence of multiple epigenetic abnormalities in cancer cells, we investigated the combined therapeutic value of 5-Aza-CdR and clorgyline to inhibit DNMTs and LSD1, in bladder cancer (T24), leukemia (HL60) and colorectal cancer (HCT116) cell lines. We observe that clorgyline employs two different mechanisms of action in the three cell lines we studied. In T24 and HL60 cells, clorgyline alone produces minimal effects on reactivation of aberrantly silenced genes as compared to the untreated control. However, combinatorial treatment induces synergistic effects on the reactivation of epigenetically silenced genes. Furthermore, only the combinatorial treatment results in enrichments of H3K4me2, H3K4me1 and H3K9/14 acetylation at the promoters of up-regulated genes. On the other hand, in HCT116 cells, clorgyline alone induces global DNA demethylation and gene reactivation. However, the combinatorial treatment did not elicit synergistic effects on gene reactivation. Despite showing different mechanisms of actions in the cell lines, collectively, our study demonstrates that combinatorial treatment has enhanced therapeutic values, and introduces a novel approach in cancer management.

## Materials and Methods

### Drug treatment and culture conditions

T24, HCT116 and HL60 cells, purchased from ATCC, were plated at 2×10^5^ cells/100-mm dish, 3×10^5^ cells/100-mm dish and 5×10^5^/25 cm^2^ flask, respectively. They were treated the next day with 1 µM, 0.3 µM or 0.1 µM of 5-Aza-CdR (Sigma-Aldrich, St. Louis, MO), respectively for 24 hours. After removal of 5-Aza-CdR, cells were treated with 10 µM clorgyline (Sigma-Aldrich, St. Louis, MO) everyday for 21 days. Multiple doses of clorgyline were tested: 1 µM, 10 µM and 100 µM. Clorgyline impaired cell growth in a dose dependent manner and the optimal dose for clorgyline (10 µM) was determined by monitoring the dose response curve.

### Colony Formation

Cells were seeded into 6-well plates at 1,000 cells per well with or with indicated treatment. The culture medium was changed every 3 days. After 10 days incubation at 37°C, the cells were washed with PBS, fixed with methanol and stained with 0.5% crystal violet. Colonies containing more than 50 cells were counted under a microscope.

### Chromatin Immunoprecipitation (ChIP) Assay

ChIP assays were performed as described previously using 4×10^6^ cells per IP [6]. Ten µg of the following antibodies were used: H3 and H3K4me2antibodies were purchased from Abcam (Cambridge, MA). Acetylated H3, and IgG antibodies were purchased from Millipore (Billerica, MA). H3K4me3 and H3K4me1 antibodies were purchased from Active Motif (Carlsbad, CA). PCR primers are available upon request.

### Real-time RT-PCR

Total RNA was isolated from cells with Trizol reagent (Invitrogen) at the indicated time points. One µg of RNA was reverse transcribed using M-MLV (Invitrogen) and random hexamers (Invitrogen). PCR reactions were performed using KAPA SYBR® FAST Universal 2X qPCR Master Mix and on Bio-Rad CFX^©^ 96 Real time PCR detection systerm. The sequences of gene specific primers are available upon request. With each set of PCR primers, titrations of known amounts of DNA were included as a standard for quantification.

### Infinium

The Infinium DNA methylation assay was performed at the USC Epigenome Center according to the manufacturer’s specifications (Illumina, San Diego, CA). The Illumina Infinium DNA methylation assay (Infinium HumanMethylation450 BeadChip) examines DNA methylation status of > 485,000 CpG sites, covering 99% of RefSeq Genes and intergenic regions selected by methylation experts. Downstream processing and beta value calculations were done as previously described [29].

### Expression Microarray

Expression analysis was performed at Sanford-Burnham Medical Institution (La Jolla, CA) using the Illumina genome-wide expression BeadChip (HumanHT-12_V4_0_R1) (Illumina). Gene expression data were processed using the lumi package in R. The data were log2 transformed and normalized using Robust Spline Normalization (RSN) as implemented in the lumi package. Comparisons between the control and treated samples for all three cell lines were performed using the R package limma. Genes (transcripts) with a p value below 0.01 and a fold-change greater than 2 relative to the control were considered significant. Oncomine™ (Compendia Bioscience, Ann Arbor, MI) was used for analysis and visualization of publicly available gene expression data. Downstream network and ontology analysis was performed using MetaCore from GeneGo Inc.

### Statistical Analysis

All statistical tests were done using R software (R version 2.15.2 , R Development Core Team, 2012). ‘Lumi’ package was used to normalize and process gene expression data. Annotation and visualization of DNA methylation probes were done using packages available through Bioconductor ("TxDb.Hsapiens.UCSC.hg19.knownGene", "rtracklayer", "Gviz" and "IlluminaHumanMethylation450kprobe"). The following CRAN packages were used to generate plots: "ggplot2", "gplots" and "VennDiagram". Gene Ontology analyses were done using the DAVID Functional Annotation Tool (Huang da et al. 2009). ChIp data were analyzed using student t tests on GraphPad Prism version 5(GraphPad Software).

### GEO Accession Number

All genome-wide data utilized in the study have been deposited in GEO under the accession number GSE41754.

## Results

### Combinatorial treatment of a DNMTi and a LSDi results in more inhibition of cell growth and colony formation than either of the single agents

**Figure 1:**
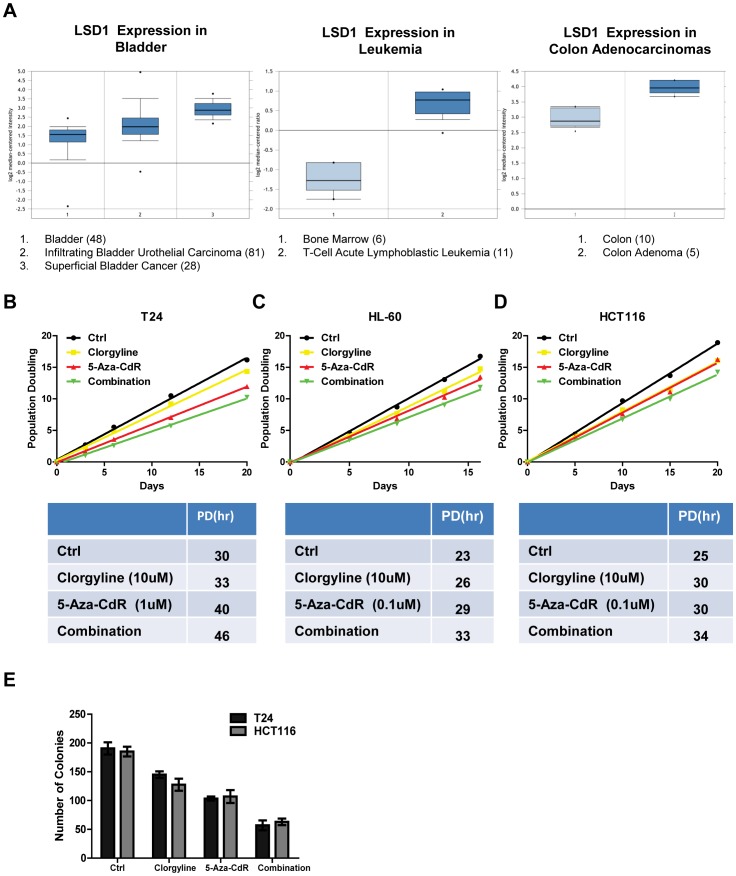
Combinatorial treatment shows the greatest efficacy in inhibiting cell growth and limiting colony formation. A. The expression levels of LSD1 in bladder cancers, T-cell acute lymphoblastic leukemia, colon adenoma and their normal counterparts were obtained from ONCOMINE. Numbers in the parentheses indicate number of samples used to generate box plots. B-D. Population doubling times for control (black), clorgyline treated (yellow), 5-Aza-CdR treated (red) and combinatorial treatment treated (green) in T24, HL60 and HCT116 cells. The corresponding numbers of hours are listed in the table below each graph. E. Quantification of number of colonies produced by colony formation assays in T24 and HCT116 cells after indicated treatments.

On analysis of gene expression data available through Oncomine™ (Compendia Bioscience, Ann Arbor, MI), we observed that LSD1 is over-expressed in bladder cancer, leukemia and colon adenocarcinomas as compared to their normal counterparts (Figure 1A), suggesting that overexpression of LSD1 may play a role in tumorigenesis. In addition, it has been well established that aberrant DNA methylation is involved in the initiation as well as the progression of various malignancies, including in the three cancers mentioned above [30]. Therefore, a bladder cancer cell line, T-24, a leukemia cell line, HL60 and a colon cancer cell line, HCT116 were chosen to investigate the effects of inhibiting LSD1 as well as to assess the therapeutic efficacy of a combinatorial treatment consisting of a LSD1i and a DNMTi.

All cell lines were treated as follows: clorgyline alone, 5-Aza-CdR alone, and combinatorial treatment of clorgyline and 5-Aza-CdR. Cells were treated with 5-Aza-CdR for 24 hours prior to changing the media. In contrast, cells received a fresh dose of clorgyline everyday for 21 days. In all three cell types examined, both 5-Aza-CdR and clorgyline treatment alone extended cell population doubling time between 3 and 10 hours, with the combinatorial treatment further increasing doubling time, up to 16 hours (Figure 1B, C, D).

To assess the therapeutic efficacy of clorgyline, 5-Aza-CdR and the combinatorial treatment on the survival and proliferation of cells, we performed colony formation assays on the two adherent cell lines, T-24 and HCT116 cells. Clorgyline showed a moderate effect in suppressing the ability of T24 and HCT116 cells to form colonies. 5-Aza-CdR substantially suppressed colony formation and the combinatorial treatment further reduced the number of colonies (Figures S1 and 1E). Our data demonstrate that both clorgyline and 5-Aza-CdR inhibit cell growth and suppress colony formation. In addition, the combinatorial treatment further slows population doubling time and reduces the ability of cells to proliferate.

### Combinatorial treatment of a DNMTi and a LSDi significantly upregulates more genes than either of the single agents in T24 and HL60 cells

**Figure 2:**
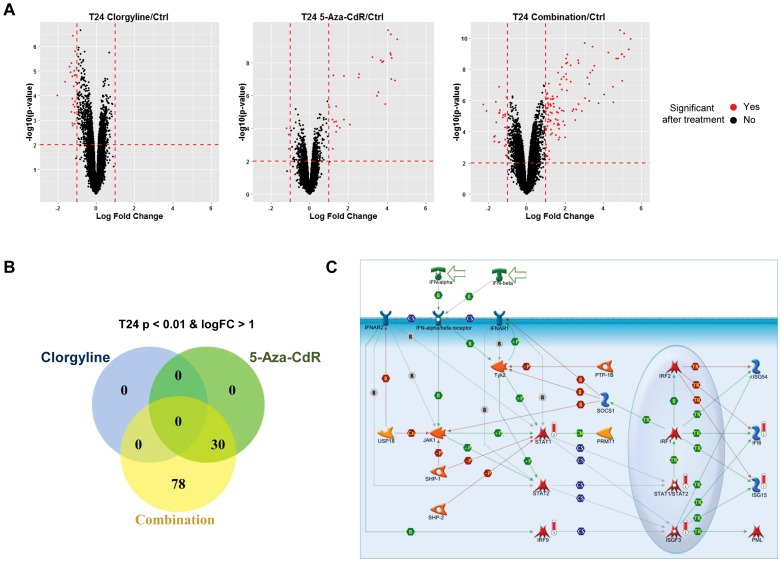
Combinatorial treatment elicits a synergistic effect in up-regulating genes in T24 cells . A. Gene expression log2 difference is plotted on the x-axis, and the –log10 (p-value) is plotted on the y-axis. Probes that are identified as significantly different between two groups are colored in red. B. Venn intersects of the number of genes that are up-regulated by the indicated treatment. C. Network enrichment diagram containing genes that are synergistically up-regulated upon combinatorial treatment. Synergistically upregulated genes are marked by red bars. The other symbols used are as seen on the Metacore website.

To investigate the global change in gene expression profile after clorgyline, 5-Aza-CdR and combinatorial treatment, we conducted genome-wide expression studies at day 18 post-treatment (D18), using the Illumina HumanHT-12 V4 BeadChip in T24 and HL60 cells. It has been well documented that 5-Aza-CdR induces immediate demethylation and subsequent gene reactivation [31], [32]. It has also been established that methylation rebounds and genes become re-silenced upon drug withdrawal [33], since DNMT levels get replenished. Therefore, we chose a relatively late time point to assess whether the combinatorial treatment can induce sustained gene reactivation. Gene expression changes of all transcripts after indicated treatments are shown as volcano plots (Figure 2A). At day 18, in T24 cells, clorgyline alone did not significantly up-regulate any transcripts, while 5-Aza-CdR treatment increased the expression of 30 transcripts. Remarkably, the combinatorial treatment induced substantially more transcripts (108 transcripts) which were significantly up-regulated (Figure 2A). To study the overlap between the up-regulated transcripts upon the different treatments we generated a Venn diagram, which shows that all the transcripts induced by 5-Aza-CdR were also induced by the combinatorial treatment (Figure 2B). Next, to compare the global gene expression induction difference between 5-Aza-CdR and the combinatorial treatment, we plotted the observed Log2 fold change for all the interrogated transcripts on the platform. The majority of those transcripts are synergistically up-regulated upon combinatorial treatment (Figure S2), showing that clorgyline supplements the ability of 5-Aza-CdR to reactivate genes in T24 cells. In addition to supplementing 5-Aza-CdR, combinatorial treatment can also reactivate genes that failed to be up-regulated by 5-Aza-CdR alone. Seventy-eight genes were only up-regulated upon combinatorial treatment (Figure 2B), suggesting that the combinatorial treatment may employ a different mechanism of action than the treatment of 5-Aza-CdR alone. Interestingly, the genes that are synergistically up-regulated upon combinatorial treatment are enriched for specific functions, such as immune response, cytoskeleton remodeling and cell cycle regulation (Figure 2C, Figure S3). A detailed analysis was done on genes which are part of the IFN alpha and IFN beta signaling pathway. The analysis revealed that the combinatorial treatment stimulates the expression of several genes which are essential for effective immune response to viral infections (Figure 2C).

**Figure 3:**
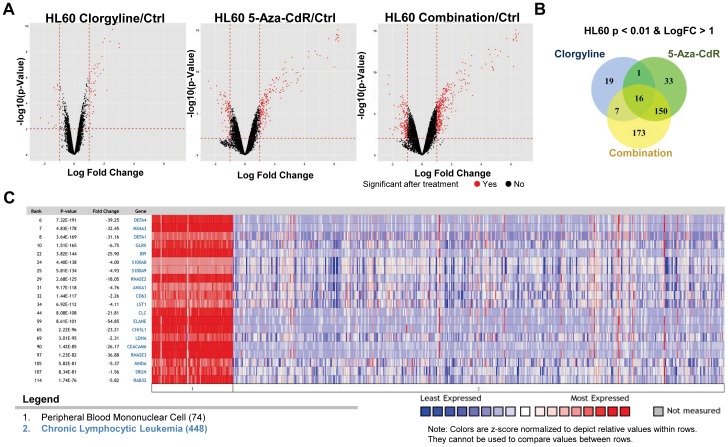
Combinatorial treatment elicits a synergistic effect in up-regulating genes in HL60 cells. A. Gene expression log2 difference is plotted on the x-axis, and the –log10 (p-value) is plotted on the y-axis. Probes that are identified as significantly different between two groups are colored in red. B. Venn intersects of the number of genes that are up-regulated upon the indicated treatment. C. Expression status obtained from ONCOMINE of 20 genes, whose expression was synergistically reactivated in HL60 cells, in peripheral blood mononuclear and chronic lymphocytic leukemia.

We also conducted genome-wide expression studies in HL60 cells. Gene expression changes of all transcripts after the indicated treatment are shown as volcano plots. Using previously established cutoffs, we identified differentially expressed transcripts, which are highlighted in red (Figure 3A). We found that while all three treatments induce gene up-regulation the combinatorial treatment induces the expression of more transcripts, exhibiting a similar expression profile to T24 cells. Clorgyline, 5-Aza-CdR and the combinatorial treatment up-regulated 43, 200 and 346 transcripts, respectively (Figure 3B). Although there was a considerable overlap between 5-Aza-CdR and the combinatorial treatment, the combinatorial treatment was more effective at gene induction in that it up-regulated 180 transcripts that were not up-regulated by 5-Aza-CdR (Figure 3B).

To assess the functional importance of the genes synergistically up-regulated by the combinatorial treatment we surveyed the Oncomine™ database (Compendia Bioscience, Ann Arbor, MI). On studying gene expression for chronic lymphocytic leukemia samples compared to normal peripheral blood mononuclear samples, we noticed that numerous genes in these categories were down-regulated in the leukemias compared to the normal cells (Figure 3C), suggesting that these genes are potential tumor suppressors in leukemia. Collectively, our data show that changes in gene expression are associated with changes in cell growth inhibition and that combinatorial treatment has more a more profound impact on inhibiting cell growth rate compared to treatment with single agents, results that are in line with our global expression studies. In addition, combinatorial treatment elicits a synergistic effect in up-regulating gene expression in both T24 and HL60 cells. Furthermore, many of those genes have potential implications for immunotherapy, such as cancer testis antigen genes, and genes in the interferon pathway, suggesting the feasibility of combining epigenetic therapy and immunotherapy in treating malignancies. Notably, our data also indicates that changes in gene expression are consistent with cell growth inhibition; the combinatorial treatments up-regulates substantially more genes, that are potentially tumor suppressive, thus resulting in the greatest impact on cell growth rate among all the treatments.

### Gene up-regulation induced by combinatorial treatment is due to altered histone modifications not demethylation in T24 cells

**Figure 4:**
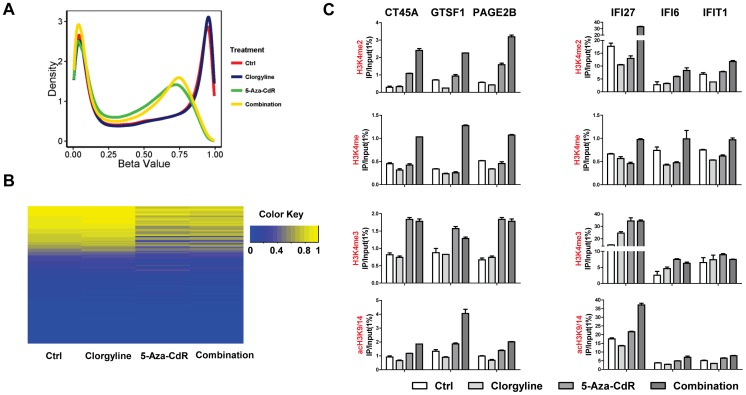
Gene up-regulation induced by combinatorial treatment is due to histone modifications in T24 cells. A. Density plots for each treatment across all 450,000 CpG sites. The x-axis represents beta values ranging from 0 (not methylated) to 1 (highly methylated). B. Heatmap of CpG probes belonging to genes which are synergistically up-regulated upon combinatorial treatment. The level of DNA methylation for each probe in each sample is represented by using the color scale shown in the legend. C. ChIP results of histone modifications after normalization to input. Error bars represent standard deviation from 3 independent experiments.

To investigate whether DNA demethylation was responsible for the up-regulation of more genes upon combinatorial treatment, we conducted global DNA methylation studies using Illumina Infinium HumanMethylation450 platform, which includes more than 450,000 CpG sites, covering promoter regions, 5'UTR, 3'UTR, gene body and first exons. Since both T24 and HL60 showed synergistic gene up-regulation due to combination treatment, we selected T24 as the representative cell line to study the cause of gene induction. The DNA methylation level for each interrogated CpG site is reported as a beta value, ranging from 0 (not methylated) to 1(fully methylated). A density plot, which includes all the probes on the array, was generated to show the global DNA methylation profiles for every treatment (Figure 4A). The probes can be roughly separated into two groups in the control based on the bimodal distribution of the beta values: a hypomethylated group (beta value<0.2) and a hypermethylated group (beta value>0.8) (Figure 4A). The control and clorgyline treated cells exhibited very similar methylation profiles. After, 5-Aza-CdR treatment, the peak representing hypermethylated probes was shifted towards lower beta values, illustrating that 5-Aza-CdR induces global demethylation. T24 cells exposed to 5-Aza-CdR treatment showed a very similar methylation profile as the cells exposed to combinatorial treatment (Figure 4A). Although DNMT1 has been reported as a potential substrate for LSD1 [16], our data show that clorgyline does not induce demethylation in T24 cells.

Next, we investigated the methylation levels of genes that were significantly up-regulated upon combinatorial treatment. A heatmap was generated to illustrate the methylation levels of probes, which are located within the promoter regions (probes located within 1500 bps of the transcription start site) of those significantly altered genes after each treatment (Figure 4B). The control and clorgyline treated cells showed very similar methylation profiles with few probes which either gained or lost methylation after clorgyline treatment (delta beta value >0.2). However, combinatorial treatment did not induce further demethylation of those probes which were hypermethylated originally. Comparing 5-Aza-CdR treatment with combinatorial treatment, 0 probes had a delta beta value greater than 0.2, confirming that there was no substantial change in methylation due to the combinatorial treatment (Figure 4B). In an unbiased manner we selected two genes, IFI27 and PAGE2B, which were synergistically reactivated by combinatorial treatment, to study changes in DNA methylation after each treatment (Figure S4 A and B). Both genes have several probes on the Infinium platform. The promoter of IFI27 is unmethylated; on the other hand, the promoter of PAGE2B is methylated. As the data demonstrates, these two genes showed similar DNA methylation levels after either combinatorial treatment or 5-Aza-CdR treatment. Taken together, our data show that genes which are significantly up-regulated upon combinatorial treatment lack the corresponding demethylation change as compared to cells treated with 5-Aza-CdR alone, suggesting it activates genes independent of DNA methylation changes in T24 cells.

To explore other potential mechanism for further gene reactivation induced by the combinatorial treatment, we decided to investigate changes in histone marks that could be associated with the observed expression changes. We first randomly selected 8 genes, irrespective of methylation status in the control, (CT45A4, GTSF1, TKTL1, PAGE2B, IFI6, IFI27, IFIT1 and HIST1H2BK) from the pool of 92 genes, which were significantly up-regulated upon combinatorial treatment, and validated our expression array results by RT-PCR at indicated days (Figure S5).

Upon confirmation of the array results, we selected 6 out of the 8 genes and investigated the changes in specific histone marks: H3K4me1, H3K4me2 and H3K4me3 by performing ChIP assays at the promoters of these genes. Both H3K4me1 and H3K4me2 have been reported as direct substrates of LSD1 [9]. Among the genes we selected, promoters of three of these genes, CT45A4, GTSF1 and PAGE2B, are methylated in T-24 cells and the rest are unmethylated. As compared to the control, no enrichments of H3K4me1 and H3K4me2, LSD1 targets, were observed at the promoters of all selected genes upon clorgyline treatment (Figure 4C). Both 5-Aza-CdR treatment and the combinatorial treatment induced enrichments of H3K4me1 and H3K4me2 at the promoters of methylated genes (Figure 4C). The enrichment levels were substantially higher upon combinatorial treatment, suggesting clorgyline supplements 5-Aza-CdR to establish an active chromatin structure at methylated genes.

At the promoters of unmethylated genes, neither 5-Aza-CdR nor clorgyline alone increased the enrichment of active chromatin marks; however, the combinatorial treatment further induced the enrichments of H3K4me2 and H3K4me1. We also examined the level of H3K4me3, which is not a direct target of LSD1, but is an active histone mark, enriched at the promoters of unmethylated genes. Clorgyline induced most dramatic enrichment of H3K4me3 for IFI27 compared to the control. 5-Aza-CdR treatment and the combinatorial treatment showed comparable enrichments of H3K4me3 for all selected genes, reinforcing that gene reactivation was induced by enrichments of H3K4me2 and H3K4me1, the two direct targets of LSD1. In addition, we investigated the enrichments of H3K9/14 acetylation because they tightly correlate with gene expression [34]. We observed that clorgyline treatment did not alter the level of acetylation as seen in the control, consistent with observed expression levels. 5-Aza-CdR induced the enrichment of acetylation and the combinatorial treatment further increased the enrichment level at the promoters of methylated genes as well as IFI27 (Figure 4C). Collectively, our results show that in T-24 cells, the combinatorial treatment induces further gene reactivation not through DNA demethylation. Of interest, it enrichments of H3K4me2 and H3K4me1 are the key activating changes that result in gene reactivation.

### Clorgyline induces DNA demethylation in HCT116 cells

**Figure 5:**
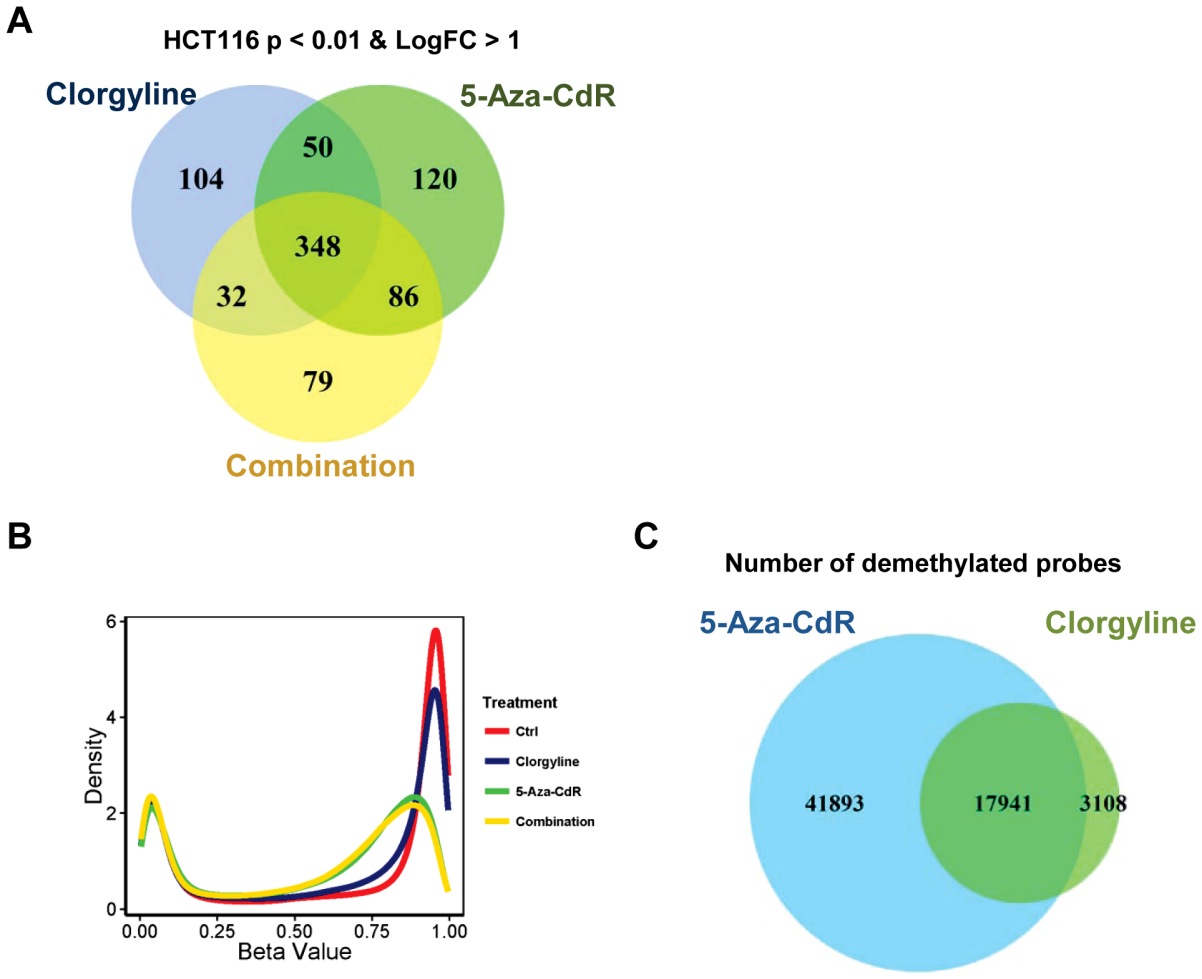
Clorgyline induces demethylation in HCT116 cells. A. Venn intersects of the genes that are up-regulated upon the indicated treatment. B. Density plots for each treatment across all 450,000 CpG sites. The x-axis represents beta values ranging from 0 (not methylated) to 1 (highly methylated). C. Venn intersect of the probes that are demethylated by the indicated treatment. The overlap of two circles represents the common probes that are demethylated by both treatments.

We conducted genome-wide expression analysis at day 20 post treatment in HCT116 cells to investigate the changes in global gene expression after each treatment. Interestingly, all three treatments: clorgyline, 5-Aza-CdR and the combinatorial treatment, induced substantial gene up-regulation as well as down-regulation (Figure S6). There is a substantial overlap (348 transcripts) among all three treatments (Figure 5A), suggesting that all the treatments may employ similar mechanisms of action in reactivating silenced genes. This may also explain the lack of synergistic response in HCT116 cells.

Although clorgyline does not induce DNA demethylation in T24 cells, we investigated whether the significant gene up-regulation observed upon all three treatments in HCT116 was attributed to DNA demethylation by conducting global DNA methylation analysis at day 20 post-treatment. The density plot demonstrates that both 5-Aza-CdR treatment and the combinatorial treatment induced substantial demethylation, exhibiting similar global methylation profiles (Figure 5B). In contrast to T24 cells, clorgyline induced moderate DNA demethylation in HCT116. At day 20, 21,049 probes were demethylated by clorgyline treatment, while 59,834 probes remained demethylated after 5-Aza-CdR treatment. There is a substantial overlap between demethylated probes between these two treatments (Figure 5C). Clorgyline primarily demethylates non-CpG island regions (Figure S7A). Since HCT116 cells have higher methylation levels in non-CpG island regions than in T24 cells (Figure S7B), this may provide an explanation for its efficacy in HCT116. It has been shown that DNMT1 is a substrate for LSD1 and LSD1 inhibitors have the potential to destabilize DNMT1, resulting in global demethylation [16]. Our laboratory has previously shown that DNMT1 is more prominent in maintaining non-CpG island methylation [35], reinforcing our hypothesis that clorgyline induces demethylation by destabilizing DNMT1. Collectively, our results demonstrate that in HCT116, clorgyline induces global DNA demethylation and gene up-regulation, suggesting that clorgyline employs different mechanisms of action in HCT116 cells than in T24 and HL-60 cells.

## Discussion

The discovery of the histone lysine demethylase, which plays an important role in gene regulation by modulating histone tail modifications and the stabilities of non-histone substrates, has opened an exciting new avenue for epigenetic therapy. Although targeting histone demethylase to reactivate aberrantly silenced genes is still in its infancy, a number of preclinical studies have shown promising outcomes. Zhu et al reported polyamine analog inhibitors of LSD1 reactivated aberrantly silenced genes by establishing an active chromatin structure [26].

Recently, a number of FDA-approved drugs targeting monoamine oxidases (MAO) have been utilized to target LSD1 *in vivo*, due to the structural homology of the catalytic domains shared by the two proteins. A number of studies reported that such inhibitors are capable of inhibiting cancer cell proliferation [25], [36], and reactivating aberrantly silenced genes and pathways [37]. However, under many circumstances, LSD1 inhibitors alone only have minimal to modest therapeutic efficacies, which are greatly enhanced in combination with other therapies [38]. Huang et al showed that re-expression of SFRP2, a negative regulator of Wnt signaling, upon co-treatment with low doses of LSD1-inhibiting oligoamine analogues and DNA methyltransferases inhibitors is much more substantial than any of the single treatment [38]. Our study further assesses the enhanced therapeutic value of combining 5-Aza-CdR and clorgyline genome-wide, using three cell lines as model systems. One advantage of introducing a combinatorial treatment consisting of two FDA-approved drugs is the relative rapidity of proceeding to clinical settings.

LSD1 has a number of substrates, including both histone and non-histone substrates [38], [39], and has been suggested to have multiple mechanisms of action to regulate gene expression [9], [28]. Our study suggests that clorgyline employs different mechanisms of action in T24 and HCT116 cells. In T24 cells, clorgyline does not induce demethylation, but rather supplements 5-Aza-CdR in the combinatorial treatment to further reactivate genes by enriching H3K4me2 and H3K4me1. Clorgyline alone has minimal effects in terms of reactivating silenced genes and establishing an active chromatin configuration. On the other hand, in HCT116 cells, clorgyline induced DNA demethylation and gene reactivation, play a similar role as those induced by 5-Aza-CdR; however, it did not result in a synergistic effect in gene up-regulation when combining with 5-Aza-CdR. This may be attributed to the difference in total methylation levels in the two cell lines. But, in both cell lines, clorgyline treatment alone showed modest cell growth inhibition, which might be attributed to its effects on non-histone protein targets, such as p53[17].

Our group has reported up-regulation of cancer-testis antigens (CTAs) as well as genes in the interferon pathway upon 5-Aza-CdR treatment in T24 cells [40]. In this study, our gene ontology results reveal that same groups of genes, CTAs and genes in the interferon pathway, were substantially up-regulated upon the combinatorial treatment, suggesting their potential implications in immunotherapy. CTA genes are normally silenced in non-germline normal tissues by DNA methylation and are only expressed in germ cells and trophoblast tissues, and are also aberrantly expressed in a variety of cancers [41]. It has been well documented that epigenetic regulation plays a vital role in regulating transcription of CTA genes, which have been actively pursued as targets for cancer vaccines [42]. However, such vaccines often yield limited therapeutic values due to the heterogeneous expression of CTA genes in tumors. Only a small fraction of cancer cells express CTA genes [42], [43]. Epigenetic therapies can robustly augment the expression of CTA genes, suggesting potentially favorable outcomes upon combining epigenetic therapies and CTA-directed therapies for cancer treatment. In addition, one can posit that amplifying the immunogenicity of tumor cells can also increase the likelihood of tumor cells to recognition and elimination by the host immune system [43].

In addition to the augmentation of CTA genes, our gene ontology results also reveal that another group of transcripts, belonging to the interferon (IFN) pathway, were substantially up-regulated by the combinatorial treatment. It is well-known that IFN signaling plays a vital role in recognition and elimination of cancer cells, thus promoting antitumor responses and protecting the host against the development of cancer [44]. Systemic injections of IFNs are approved for the treatment of both solid and hematological malignancies [45]. A subclass of IFN has been used in combinatorial therapy of glioblastoma [46]. Epigenetic therapy can augment and sustain the expression of IFNs, which can induce multiple downstream anti-tumor actions, offering opportunities to develop novel combinatorial therapies.

In summary, our study demonstrates that a combinatorial treatment consisting of a DNMT inhibitor and a LSD1 inhibitor results in enhanced cell growth inhibition and a synergistic effect in up-regulating gene expression in both T24 and HL60 cell lines. Those up-regulated genes may play a key role in making cancerous cells less tumorigenic, reflected by slower growth rate and limited ability to form colonies. In addition, combinatorial treatment augments the expression of a class of genes belonging to certain immune-related pathways, which have the potential to prime cells for further immunotherapy. Furthermore, we show that clorgyline has two potential mechanisms of action. Which mechanism dominates is cell line dependent.

## Supporting Information

Figure S1 **Combinatorial treatment results in greatest inhibition of colony formation.** Images of plates used to test the different treatments by colony formation assay demonstrate that clorgyline had the most significant impact on colony growth.
(PDF)

Figure S2 **Combinatorial treatment up-regulates more transcripts than 5-Aza-CdR.** Scatter plot of the log fold change induced by 5-Aza-CdR treatment versus the log fold change induced by the combination treatment for all transcripts. Red dots represent transcripts that are differentially expressed upon combinatorial treatment.
(PDF)

Figure S3 **Combinatorial treatment up-regulates genes that have specific functions.** Gene ontology results show enrichments of specific functions in the genes that are up-regulated upon the combinatorial treatment.
(PDF)

Figure S4 **Schematic diagrams for locations of probes on the Infinium platform.** A, B. The positions of each vertical bar next to the indicated treatments represent the locations of each probe. Yellow represents fully methylated; blue represents unmethylated. Arrows indicate the direction of transcription.
(PDF)

Figure S5 **Validations of genome-wide expression array results.** RT-PCR results showing the gene expression levels at both D12 and D18 after indicated treatments. The mRNA levels were normalized to GAPDH. Error bars represent the standard deviation of biological triplicates.
(PDF)

Figure S6 **All three treatments induced substantial gene up-regulation as well as down-regulation.** Gene expression log2 difference is plotted on the x-axis, and the –log10 (p-value) is plotted on the y-axis. Probes that are identified as significantly different between two groups are colored in red.
(PDF)

Figure S7 **Differential DNA demethylation upon drug treatment.** A. Blue indicates CpG island probes, which were demethylated. Red indicates the percentage of non-CpG island probes, which were demethylated. B. Blue bars indicate methylated CpG island probes; red bars indicate methylated non-CpG island probes. C. Density plots for total methylation in T24 and HCT116 cells across all 450,000 CpG sites. The x-axis represents beta values ranging from 0 (not methylated) to 1 (highly methylated). Blue line represents T24 cells; red line represents HCT116 cells.
(PDF)
