# Supplementary figures and images for: Synergistic Re-Activation of Epigenetically Silenced Genes by Combinatorial Inhibition of DNMTs and LSD1 in Cancer Cells
Source: PLoS One. 2013 Sep 6;8(9):e75136. doi: 10.1371/journal.pone.0075136 (PMC3765366; doi:10.1371/journal.pone.0075136)

**Figure S1**

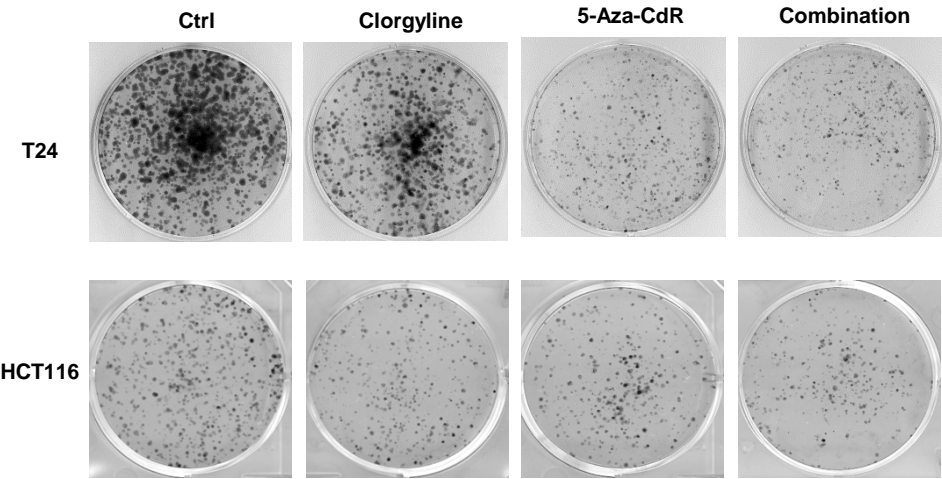

Supplement: Figure S1 — Combinatorial treatment results in greatest inhibition of colony formation. Images of plates used to test the different treatments by colony formation assay demonstrate that clorgyline had the most significant impact on colony growth. (PDF) [file pone.0075136.s001.pdf]

Figure S2

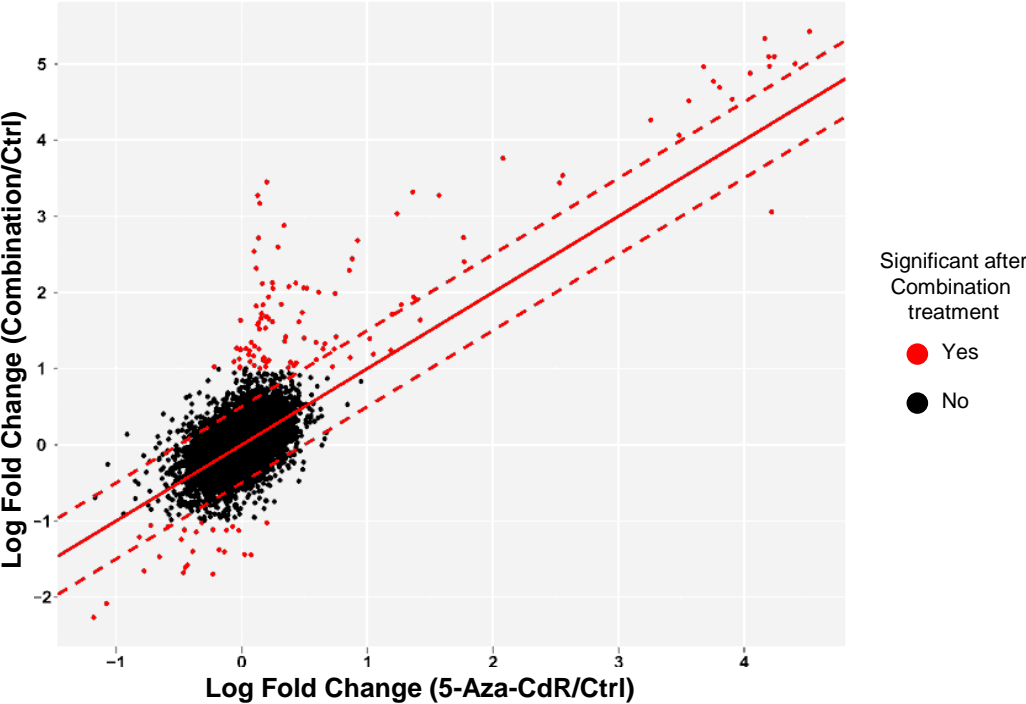

Supplement: Figure S2 — Combinatorial treatment up-regulates more transcripts than 5-Aza-CdR. Scatter plot of the log fold change induced by 5-Aza-CdR treatment versus the log fold change induced by the combination treatment for all transcripts. Red dots represent transcripts that are differentially expressed upon combinatorial treatment. (PDF) [file pone.0075136.s002.pdf]

**Figure S3**

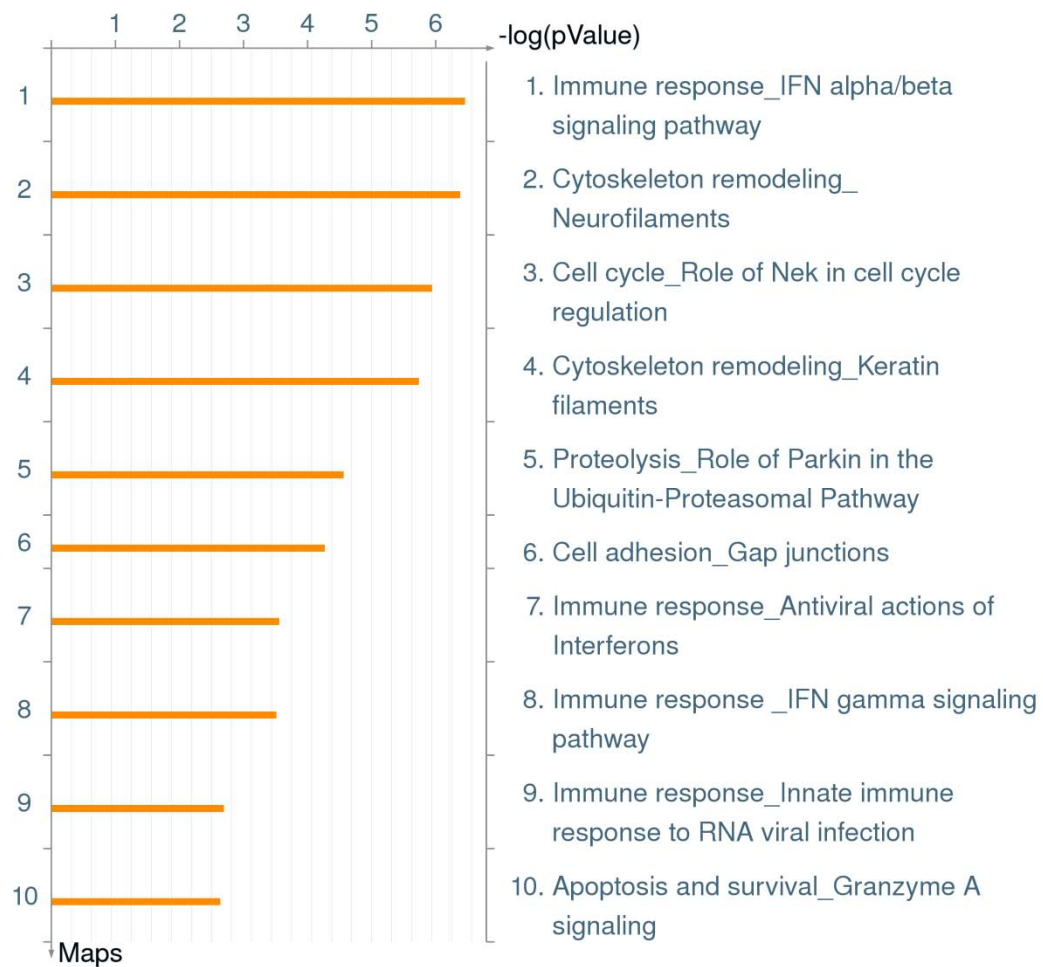

Supplement: Figure S3 — Combinatorial treatment up-regulates genes that have specific functions. Gene ontology results show enrichments of specific functions in the genes that are up-regulated upon the combinatorial treatment. (PDF) [file pone.0075136.s003.pdf]

Figure S4

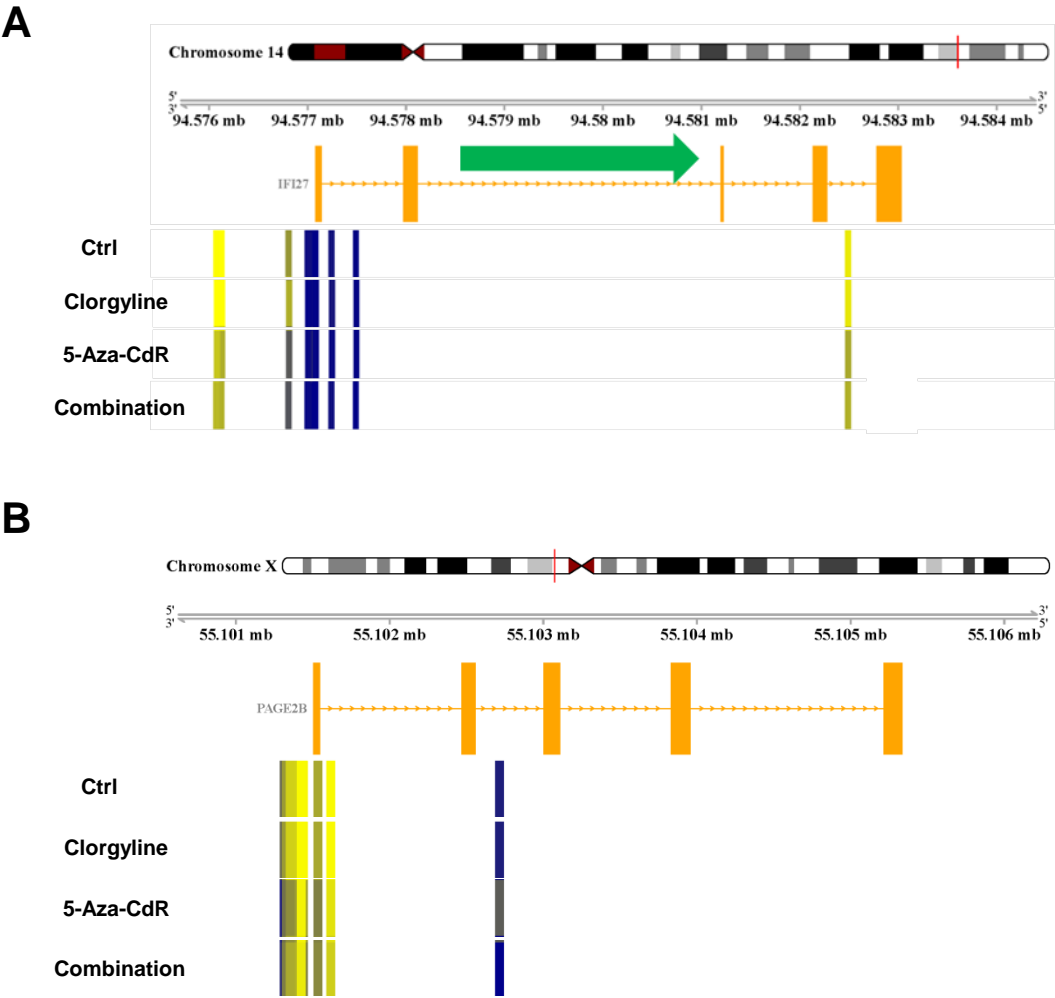

Supplement: Figure S4 — Schematic diagrams for locations of probes on the Infinium platform. A, B. The positions of each vertical bar next to the indicated treatments represent the locations of each probe. Yellow represents fully methylated; blue represents unmethylated. Arrows indicate the direction of transcription. (PDF) [file pone.0075136.s004.pdf]

Figure S5

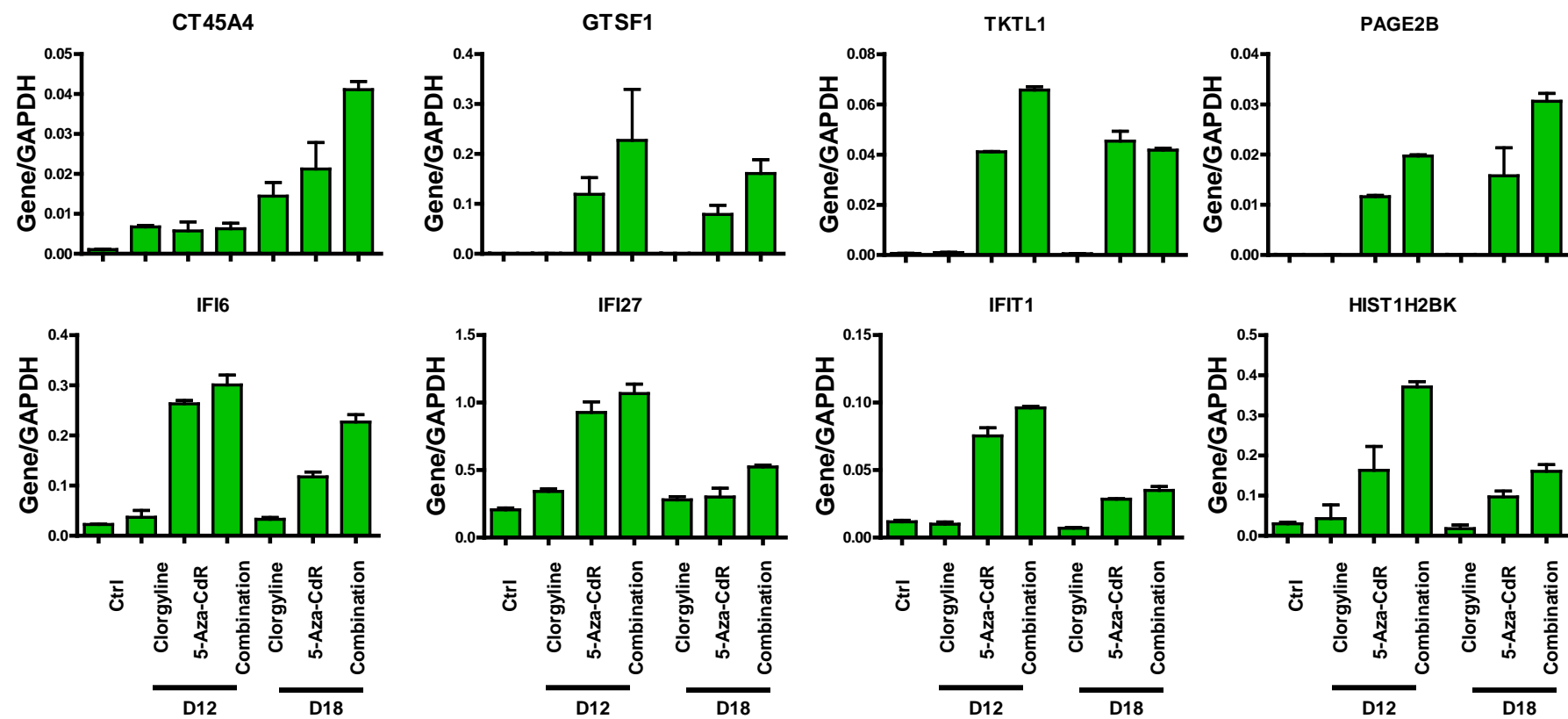

Supplement: Figure S5 — Validations of genome-wide expression array results. RT-PCR results showing the gene expression levels at both D12 and D18 after indicated treatments. The mRNA levels were normalized to GAPDH. Error bars represent the standard deviation of biological triplicates. (PDF) [file pone.0075136.s005.pdf]

Figure S6

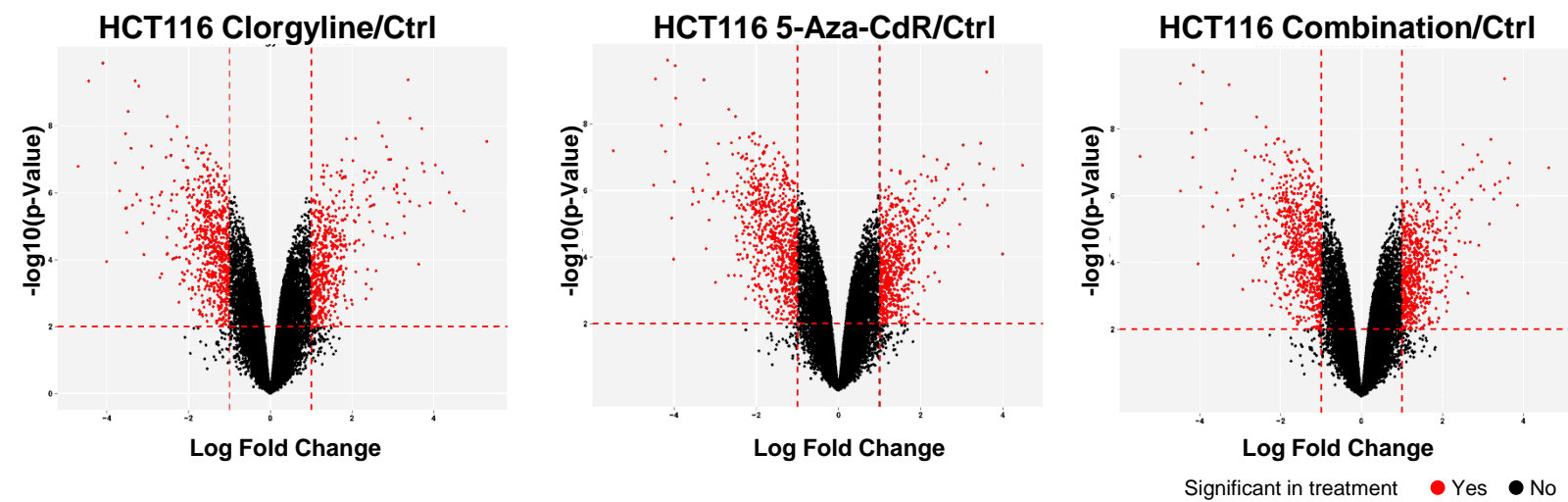

Supplement: Figure S6 — All three treatments induced substantial gene up-regulation as well as down-regulation. Gene expression log2 difference is plotted on the x-axis, and the –log10 (p-value) is plotted on the y-axis. Probes that are identified as significantly different between two groups are colored in red. (PDF) [file pone.0075136.s006.pdf]

**Figure S7**

**A**

**Clorgyline D20 Demethylated Probes**

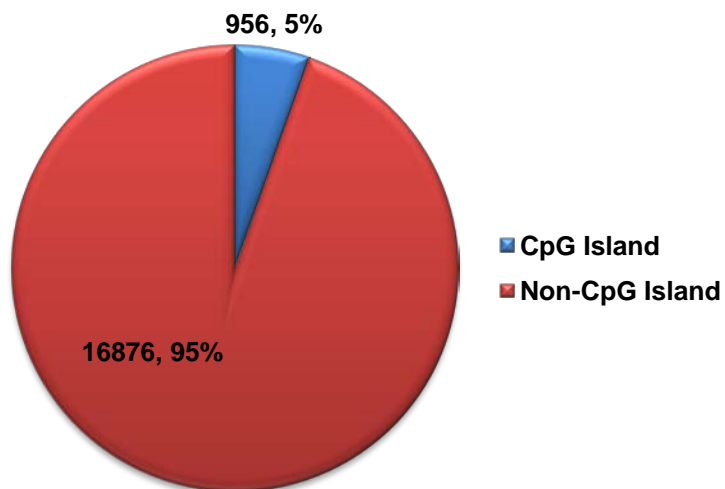

**B**

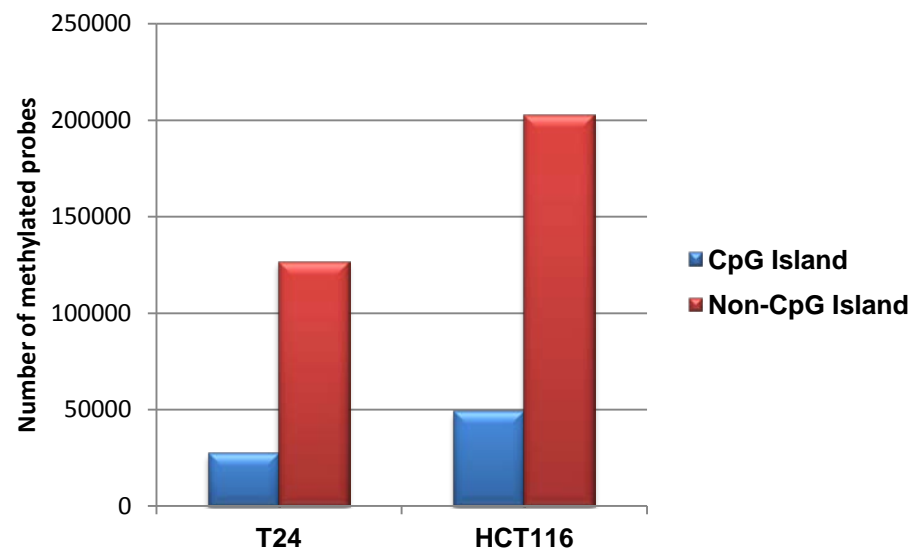

**C**

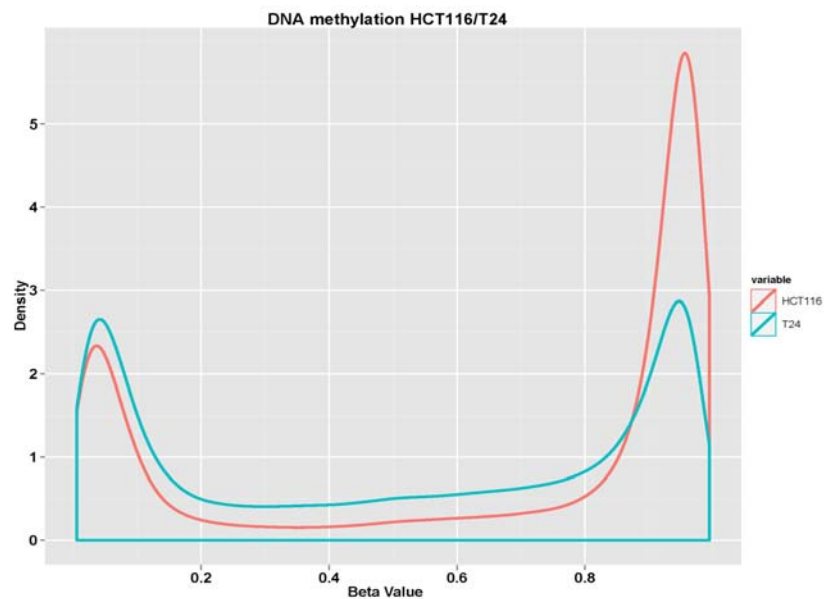

Supplement: Figure S7 — Differential DNA demethylation upon drug treatment. A. Blue indicates CpG island probes, which were demethylated. Red indicates the percentage of non-CpG island probes, which were demethylated. B. Blue bars indicate methylated CpG island probes; red bars indicate methylated non-CpG island probes. C. Density plots for total methylation in T24 and HCT116 cells across all 450,000 CpG sites. The x-axis represents beta values ranging from 0 (not methylated) to 1 (highly methylated). Blue line represents T24 cells; red line represents HCT116 cells. (PDF) [file pone.0075136.s007.pdf]
